# STAT3 Partly Inhibits Cell Proliferation *via* Direct Negative Regulation of *FST* Gene Expression

**DOI:** 10.3389/fgene.2021.678667

**Published:** 2021-06-22

**Authors:** Haidong Xu, Guangwei Ma, Fang Mu, Bolin Ning, Hui Li, Ning Wang

**Affiliations:** ^1^College of Animal Science and Technology, Northeast Agricultural University, Harbin, China; ^2^Ministry of Education Key Laboratory for Ecology of Tropical Islands, Key Laboratory of Tropical Animal and Plant Ecology of Hainan Province, College of Life Sciences, Hainan Normal University, Haikou, China

**Keywords:** STAT3, FST, transcriptional regulation, cell proliferation, sheep

## Abstract

Follistatin (FST) is a secretory glycoprotein and belongs to the TGF-β superfamily. Previously, we found that two single nucleotide polymorphisms (SNPs) of sheep *FST* gene were significantly associated with wool quality traits in Chinese Merino sheep (Junken type), indicating that FST is involved in the regulation of hair follicle development and hair trait formation. The transcription regulation of human and mouse *FST* genes has been widely investigated, and many transcription factors have been identified to regulate *FST* gene. However, to date, the transcriptional regulation of sheep *FST* is largely unknown. In the present study, genome walking was used to close the genomic gap upstream of the sheep genomic *FST* gene and to obtain the *FST* gene promoter sequence. Transcription factor binding site analysis showed sheep *FST* promoter region contained a conserved putative binding site for signal transducer and activator of transcription 3 (STAT3), located at nucleotides −423 to −416 relative to the first nucleotide (A, +1) of the initiation codon (ATG) of sheep *FST* gene. The dual-luciferase reporter assay demonstrated that STAT3 inhibited the *FST* promoter activity and that the mutation of the putative STAT3 binding site attenuated the inhibitory effect of STAT3 on the *FST* promoter activity. Additionally, chromatin immunoprecipitation assay (ChIP) exhibited that STAT3 is directly bound to the *FST* promoter. Cell proliferation assay displayed that FST and STAT3 played opposite roles in cell proliferation. Overexpression of sheep *FST* significantly promoted the proliferation of sheep fetal fibroblasts (SFFs) and human keratinocyte (HaCaT) cells, and overexpression of sheep *STAT3* displayed opposite results, which was accompanied by a significantly reduced expression of *FST* gene (*P* < 0.05). Taken together, STAT3 directly negatively regulates sheep *FST* gene and depresses cell proliferation. Our findings may contribute to understanding molecular mechanisms that underlie hair follicle development and morphogenesis.

## Introduction

The hair follicle is a skin appendage with a complex structure composed of the dermal papilla, hair bulbs, outer root sheaths (ORS), inner root sheaths (IRS), and the hair matrix (Schneider et al., 2009; Plowman and Harland, 2018). Hair follicle morphogenesis and development involve proliferation, differentiation, and apoptosis of hair follicle stem cells (Alonso and Fuchs, 2006; Plowman and Harland, 2018). The hair follicle undergoes life-long cyclic transformations exhibiting anagen (growth), catagen (regression), and telogen (rest) phases, respectively (Stenn and Paus, 2001; Schneider et al., 2009). A variety of growth factors and cytokines have been shown to tightly regulate the hair follicle morphogenesis and development through acting on the epithelial-mesenchymal interaction (Müller-Röver et al., 2001; Wang et al., 2012), such as fibroblast growth factor (FGF) (Pispa and Thesleff, 2003), tumor necrosis factor (TNF) (Liu et al., 2018) and transforming growth factor-β (TGF-β) (Li et al., 2016). As an antagonist of the TGF-β superfamily, follistatin (FST) is highly expressed in the matrix of hair follicles, which consist of cells with a strong proliferation ability (Ma et al., 2017). *FST* transgenic mice exhibited shinier and more irregular hair (Guo et al., 1998; Wankell et al., 2001). *FST* knockout mice died within hours after birth and displayed curlier whiskers (Matzuk et al., 1995; Jhaveri et al., 1998; Nakamura et al., 2003). Our previous association analysis demonstrated that two single nucleotide polymorphisms (SNPs) in sheep *FST* gene were associated with wool quality traits in Chinese Merino sheep (Junken Type) (Ma et al., 2017). Collectively, these data indicated that FST is involved in the regulation of hair follicle development and hair trait formation.

The transcriptional regulation of human and mouse *FST* genes has been widely investigated, and many transcription factors have been identified to regulate *FST* gene. For example, erythroid 2 related factor (Nrf2) directly regulates *FST* gene and inhibits the apoptosis of human lung epithelial cells and A549 cells (Lin et al., 2016). It has been shown that transcription of *FST* gene is directly regulated by β-catenin/transcription factor 4 (TCF4) transcription factor complex to promote the murid myogenic differentiation and myoblast fusion *in vitro* and *in vivo* (Han et al., 2014). *FST* gene, involving in skeletal muscle development of *L. crocea*, is suppressed by MyoD and Sox8 (Yang et al., 2016). Myogenin promotes the satellite cell differentiation of adult mouse myogenesis in an *FST-*dependent manner (Jones et al., 2015). Estrogen-related receptor β (ERRβ) inhibits epithelial to mesenchymal transition in breast cancer through directly boosting *FST* expression (Sengupta et al., 2014). Moreover, transcription factor SP1 induces *FST* transcription in intestinal epithelial cells and kidney mesangial cells (Necela et al., 2008; Mehta et al., 2019). *FST* gene expression is predominantly up-regulated by GLI family zinc finger 2 (*GLI2*) in human keratinocytes (Eichberger et al., 2008). However, to date, the transcriptional regulation of sheep *FST* is largely unknown.

In this study, we investigated the transcription regulation of sheep *FST* gene by a transcription factor, signal transducer and activator of transcription 3 (STAT3), and our results demonstrated that *FST* gene is a target of STAT3 and that STAT3 inhibits cell proliferation at least partly *via* direct negative regulation *FST* gene expression.

## Materials and Methods

### Ethics Statement

All animal experiments were conducted according to the guidance for the care and use of experimental animals established by the Ministry of Science and Technology of the People’s Republic of China (Approval number: 2006-398) and approved by the Laboratory Animal Management Committee of Northeast Agricultural University.

### Cell Culture

HEK293T and HaCaT cells, purchased from the China Center for Type Culture Collection, were cultured in Dulbecco’s Modified Eagle’s Medium (DMEM) (Gibco, United States). Sheep fetal fibroblasts (SFFs), gifted from Dr. Tiezhu An, Northeast Forestry University, Harbin, were cultured in DMEM nutrient mixture F12 (DMEM-F12, Gibco, United States). All cells were cultured in the medium supplemented with 10% fetal bovine serum (FBS) (Biological Industries, Germany) plus 1% penicillin/streptomycin (Gibco, United States) at 37°C in 5% CO_2_.

### Genome Walking

There is a genomic gap immediately upstream of the sheep *FST* gene according to *Ovis aries* reference genome (ISGC Oar_v3.1/oviAri3). To close the genomic gap upstream of the sheep FST gene, genome walking was performed as previously described (Shapter and Waters, 2014). Briefly, genomic DNA was isolated from sheep skin samples, previously collected and preserved (Ma et al., 2017), using the phenol-chloroform method (Green and Sambrook, 2014). Three *FST* gene-specific reverse primers: FST-SP1, FST-SP2, and FST-SP3, were designed and synthesized. Their sequences and location are presented in Supplementary Table 1 and Supplementary Figure 1, respectively. Three forward primers: ZFP2, ZSP1, and ZSP2 were provided by the KX Genome Walking Kit (Zomanbio, China). Three rounds of polymerase chain reaction (PCR) were performed to amplify the genomic gap region with these primers. The 3′ end of ZFP2 is a random sequence and its 5′ end is a specific sequence, which can be matched by primers ZSP1 and ZSP2 in second- and third-round PCR reactions, respectively. The first PCR (primers: ZFP2 and FST-SP1) was performed using genomic DNA as a template. Second PCR (primers: ZSP1 and FST-SP2) and the third PCR (primers: ZSP2 and FST-SP3) were performed using the product (1 μL) from the first and second rounds of PCR as a template, respectively. The first PCR was performed in a reaction volume of 50 μL including 200 ng genomic DNA, 10 μL dNTPs (2.5 mM), 25 μL 2 × Kx PCR Buffer (with Mg^2+^), 1 μL Kx Pfu DNA Polymerase (1 U/μL), 7.5 μL ZFP2primers (10 pmol/μL), and 1.5 μL *FST*-SP1 (10 pmol/μL). The first PCR conditions were as follows: initial denaturation at 94°C for 2 min, followed by 2 cycles (98°C for 10 s, 60°C for 30 s, 68°C for 2 min), 98°C for 10 s, 25°C for 2 min, 25 to 68°C for 0.2°C/s, 68°C for 2 min, 6 cycles (98°C for 10 s, 60°C for 30 s, 68°C for 2 min, 98°C for 10 s, 60°C for 30 s, 68°C for 2 min, 98°C for 10 s, 44°C for 30 s, 68°C for 2 min), with a final extension at 68°C for 5 min. The second and third PCRs were also conducted in a 50 μL reaction volume including 1 μL template, 10 μL dNTPs (2.5 mM), 25 μL 2 × Kx PCR Buffer (with Mg^2+^), 1 μL Kx Pfu DNA Polymerase (1U/μL), and 1.5 μL primers (ZSP1 and *FST*-SP2 for the second PCR, ZSP2, and FST-SP3 for the third PCR, 10 pmol/μL), and run with a thermal protocol of 94°C for 2 min, followed by 30 cycles (98°C for 10 s, 60°C for 30 s, 68°C for 2 min), with a final extension at 68°C for 5 min. The third PCR product was resolved on a 1.2% agarose gel, recovered, and cloned into pEASY-T1 Simple Cloning Vector (TransGen Biotech, China) for sequencing in both directions.

### Bioinformatics Analysis

In this study, the first nucleotide (A) of the initiation codon (ATG) of *FST* was assigned position + 1. The *FST* promoter sequences of different animal species were obtained from the UCSC website^1^. The conserved transcription factor binding sites were predicted by using the Mulan website tool^2^ with the option “optimized for function” in matrix similarity and “vertebrates” in biological species (Ovcharenko et al., 2005).

### Plasmid Construction and Transient Transfection

For the construction of *FST* and *STAT3* expression vectors, based on *FST* (NM_001257093.1) and *STAT3* (XM_015098787) sequences, two pairs of primers (FST-V and STAT3-V, Supplementary Table 1) were designed to amplify the full-length coding regions (CDSs) of sheep *FST* and *STAT3* genes, respectively. The full-length CDSs of *FST* and *STAT3* were amplified by reverse transcription PCR (RT-PCR) from the pooled total RNA of sheep skin (*n* = 3) using the primer pairs FST-V and STAT3-V, respectively. The *FST* and *STAT3* PCR products were individually ligated into the pCMV-Myc (Clontech, United States), and the resulting plasmids were named pCMV-Myc-FST and pCMV-Myc-STAT3, respectively.

To construct *FST* promoter luciferase reporter vectors, the highly conserved region (−980/−340) of sheep *FST* promoter, which harbored the conserved putative STAT3 binding site (from −423 to −416), was PCR amplified with two primer pairs FST-P(+) and FST-P(−) (Supplementary Table 1) using sheep genomic DNA as the template. Subsequently, the two amplified *FST* promoter fragments were inserted into the *Kpn*I and *Hin*dIII sites of pGL3-basic (Promega, United States) to yield two *FST* promoter reporters. The reporter with the *FST* promoter fragment in the right direction was named pGL3-FST(−980/−340) and the other one with the *FST* promoter fragment in opposite direction was named pGL3-FST(−340/−980).

There was only one putative STAT3 binding site “CGATTCCCC” in sheep FST promoter (locating from −423 to −416). The mutation of this putative STAT3 binding site was expected to prevent STAT3 from binding to the *FST* promoter (Shackleford et al., 2011). This site mutation has not been reported to be associated with the wool trait. To test whether STAT3 directly regulates sheep *FST* gene, this putative STAT3 binding site was mutated to CGAGGTACC in the reporter pGL3-FST(−980/−340) using the Fast Mutagenesis System (TransGen, China) and the primer pairs FST-M according to the manufacturer’s recommendation. The resulting reporter construct was named pGL3-FST(−980/−340)-mutSTAT3. All primers were synthesized by Invitrogen (Shanghai, China) and all constructs were confirmed by Sanger sequencing (Invitrogen, Shanghai, China).

### Dual-Luciferase Reporter Assay

Briefly, the HEK293T cells were seeded in a 24-well plate (2 × 10^5^ cells^/^well) and cultured in the DMEM medium supplemented with 10% FBS and 1% penicillin/streptomycin. After the HEK293T cell reached 70–80% confluence, HEK293T cells were co-transfected with either pGL3-basic, pGL3-FST(980/−340), pGL3-FST(−340/−980) or pGL3-FST(−980/−340)-mutSTAT3 and either pCMV-Myc or pCMV-Myc-STAT3 using Lipofectamine 2000 (Invitrogen, United States) according to the manufacturer’s instructions. Dual-luciferase reporter assays were performed 48 h post-transfection using the dual-luciferase reporter assay system (Promega, United States) according to the manufacturer’s instructions. The firefly luciferase (*Fluc*) signal was normalized to that of Renilla luciferase (*Rluc*).

### Western Blot Analysis

Western blotting was performed to identify the two expression vectors (pCMV-Myc-FST and pCMV-Myc-STAT3). Briefly, HEK293T cells were transfected with pCMV-Myc-FST or pCMV-Myc-STAT3, 48 h after transfection, the cells were harvested in RIPA lysis buffer (Beyotime, China) containing 1% phenyl methane sulfonyl fluoride (Beyotime, China). After incubation on ice for 30 min, the supernatant was collected by centrifugation at 10, 000 × g for 5 min at 4°C. Equal amounts of protein from the cell lysates were separated by 12% sulfate dodecyl sodium-polyacrylamide gel electrophoresis (SDS-PAGE) and transferred to nitrocellulose membranes (Millipore, United States). After blocking with 5% (w/v) dry milk and 0.1% Tween 20 for 2 h, the membranes were incubated with Myc-tag mouse monoclonal antibody (Abcam, 1:1,000) at room temperature for 2 h. Subsequently, the membranes were incubated with horseradish peroxidase-conjugated secondary rabbit anti-mouse IgG (H&L) antibody (Abcam, 1:5,000) at room temperature for 1 h, followed by visualization using an ECL Plus detection kit (Beyotime, China).

### Chromatin Immunoprecipitation Assay

Chromatin immunoprecipitation was accomplished using a ChIP assay kit (Beyotime, China) as previously described (Deng et al., 2012). Briefly, HEK293T cells were co-transfected with pGL3-FST(−980/−340) and either pCMV-Myc-STAT3 or pCMV-Myc, at 48 h post-transfection, the cells were fixed with 1% formaldehyde at room temperature for 10 min. The Chromatin was digested with 0.5 μL micrococcal nuclease into 100–900 bp DNA/protein fragments, following immunoprecipitated with 5 μg of anti-Myc antibody (Abcam, United States) and mouse IgG (negative control) (Beyotime, China), respectively. The purified DNA fragments were measured by quantitative PCR (qPCR) using the FST-C primer pairs (Supplementary Table 1), which was performed on the 7,500 Fast Real-Time PCR System (Applied Biosystems, United States) with SYBR Green PCR Master Mix (Roche Molecular Systems, United States). The qPCR reaction volume was 20 μL including 1 μL of cDNA, 10 μL of 2 × SYBR Green PCR MasterMix (Roche Molecular Systems, United States), 0.5 μL each of the forward and reverse primers (10 μM), and 8 μL double-distilled water. The qPCR conditions were as follows: 95°C for 10 min; 40 cycles at 95°C for 30 s, 60°C for 30 s. Non-immunoprecipitated DNA (2%) was used as input control. Two additional negative controls, mouse IgG (A) and anti-Myc antibody (B), were prepared by the co-transfection of HEK293T cells with pGL3-FST(−980/−340) and pCMV-Myc. The qPCR data were normalized to input chromatin DNA and presented as fold enrichment over the input control using ΔCt equation (Tatler et al., 2016), which signal relative to input = 0.2 × 2^–Δ^ ^*Ct*^, ΔCt = Ct_[*IPsample*]_ − Ct_[*Inputsample*]_ (Patrik, 2018). The amplification product of the qPCR was analyzed by agarose gel electrophoresis with 1.5% consistency (g/mL).

### Cell Counting Kit-8 Assay

The Cell Counting Kit-8 (CCK-8) assay was used to assay cell proliferation. Briefly, the SFFs and HaCaT cells were seeded in a 96-well plate (2 × 10^4^ cells/well) and cultured in the DMEM-F12 and DMEM medium, respectively, supplemented with 10% FBS and 1% penicillin/streptomycin. The cells were individually transfected with pCMV-Myc, pCMV-Myc-FST or pCMV-Myc-STAT3 for 24, 48, and 72 h, every well was added with 10 μL CCK-8 solution (TransGen, China) and incubated at 37°C for 2 h, following the absorbance was measured at 450 nm using a Model 680 Microplate Reader (Bio-Rad, United States). The cells transfected with pCMV-Myc were used as the negative control.

### RNA Isolation, Reverse Transcription, and qPCR

The SFFs and HaCaT cells were transfected with pCMV-Myc, pCMV-Myc-FST, or pCMV-Myc-STAT3, at 48 h after transfection, total RNAs were isolated using Trizol reagent (Invitrogen, United States) according to the standard procedures, and RNA quality was assessed by denaturing formaldehyde agarose gel electrophoresis. Reverse transcription was performed with ImProm-II Reverse Transcriptase (Promega, United States) according to the manufacturer’s protocols.

The expression of proliferation marker genes, *Ki67* and proliferating cell nuclear antigen (*PCNA*), were detected by quantitative real-time PCR (qPCR). The qPCR reaction volume was 20 μL containing 1 μL of cDNA, 10 μL of 2 × SYBR Green PCR MasterMix (Roche Molecular Systems, United States), 0.5 μL each of the forward and reverse primers (10 μM), and 8 μL double-distilled water. Thermal cycling consisted of an initial step at 95°C for 10 min followed by 40 cycles at 95°C for 30 s and 60°C for 30 s. The primers used for qPCR are listed in Supplementary Table 1. The target gene expression was normalized to the glyceraldehyde 3-phosphate dehydrogenase (*GAPDH*) gene using the 2^–Δ^ ^*Ct*^ method, where ΔCt = Ct_[*targetgene*]_-Ct_[*GAPDH*]_. The negative control was the cells transfected with pCMV-Myc.

### Statistical Analysis

Results were expressed as the mean ± standard error of the mean (SEM), and all experiments were performed in triplicate. The equity of variance was tested using the Hartley F test and the result showed that the variance was homogeneous. In our present study, every group had three samples, which was not enough for normality testing. Because of the continuity and regularity of gene expression, we considered that the data were normally distributed. The statistical significance of differences was evaluated with the student’s *t*-test using SAS 9.1.3 (SAS Institute lnc., NC). Statistical significance was indicated by ^∗^*P* < 0.05, ^∗∗^*P* < 0.01, or different letters above error bars indicating a statistical significance (*P* < 0.05).

## Results

### Sheep FST Promoter Contains a Conserved Putative STAT3 Binding Site

**FIGURE 1:**
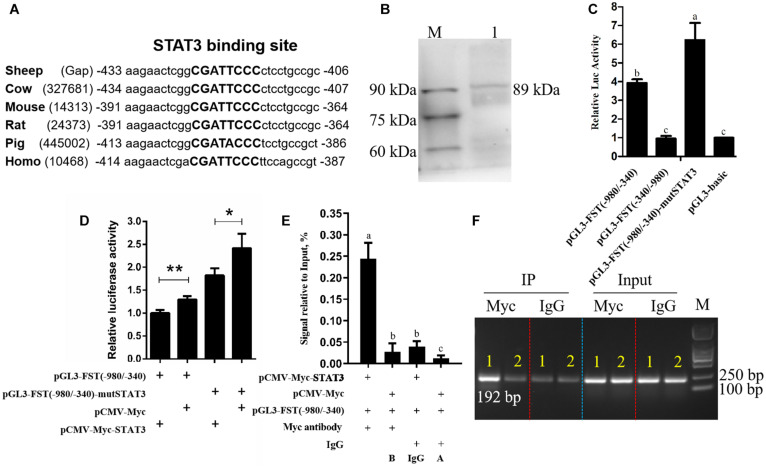
Effects of STAT3 overexpression on *FST* gene promoter activity. **(A)** Conservation analysis of STAT3 binding sites in *FST* promoter among various species, STAT3 binding sites are announced by capital letters and their locations are relative to the first nucleotide (A, + 1) of the initiation codon (ATG) of the *FST* gene. **(B)** Western blot recognition of the STAT3 expression vector (pCMV-Myc-STAT3). Lane 1: the lysate of the cells transfected with pCMV-Myc-STAT3 (89 kDa); M: Protein markers (25 kDa-90 kDa). **(C)** The effect of the mutation of STAT3 binding site on *FST* promoter activity. **(D)** Effects of STAT3 on *FST* gene promoter activity. **(E)** ChIP-qPCR analysis of the binding of STAT3 to the *FST* promoter. **(F)** The agarose gel electrophoresis analysis of ChIP-qPCR products. Lane 1: The qPCR products generated from the immunoprecipitated DNA which was isolated from the cells co-transfected with pCMV-Myc-STAT3 and pGL3-FST(–980/–340); Lane 2: The qPCR products generated from the immunoprecipitated DNA which was isolated from the cells co-transfected with pCMV-Myc and pGL3-FST(–980/–340). All data are representative of three independent experiments and display as the mean ± SEM. For each figure layer, statistical significance was indicated by *P < 0.05, **P < 0.01, and different letters above error bars indicated a statistical significance (*P* < 0.05).

There is a genomic gap immediately upstream of the sheep *FST* gene according to *Ovis aries* reference genome (ISGC Oar_v3.1/oviAri3). To obtain the promoter sequence of the sheep *FST* gene, we first closed the genomic gap by genome walking. The results showed that the gap sequence was 775 bp in length and we submitted the sequence to GenBank (Accession No. MT917184). The complete genomic sequence immediately upstream of the initiation codon (ATG) of the sheep *FST* gene was obtained by sequence assembly using the acquired genomic gap sequence and the genomic sequence from the UCSC Genome Browser database (see text footnote 1). Then we performed sequence alignment of *FST* promoters (3-kb genomic sequences immediately upstream of the initiation codon (ATG) of *FST* genes) from various animal species, which were obtained from the UCSC Genome Browser database (see text footnote 1), including sheep, cow, pig, human, mouse, and rat. The result showed that there was a conserved region, which was located at the −1,900/−1 region of sheep *FST* gene promoter. Using Mulan website tool (see text footnote 2), several putative binding sites for transcription factors, such as homeobox A4 (HOXA4), E2F transcription factor 2 (E2F2), hepatocyte nuclear factor 4 (HNF4), and STAT3, were predicted within the conserved region of the sheep *FST* gene promoter. As shown in Figure 1A, sequence alignment showed that a putative STAT3 binding site was conserved among various animal species. Interestingly, STAT3 interested us. STAT3 belongs to the signal transduction and transcription activation factor family in cell signal activation and transduction (Kong et al., 2019). It has been shown that STAT3 plays a vital role in the hair follicle and morphogenesis and development (Sano et al., 2008; Yu et al., 2014; Nelson et al., 2015; Gong et al., 2018, 2020). Whether STAT3 regulates *FST* is not clear.

### STAT3 Inhibits the FST Promoter Activity

To test the hypothesis that STAT3 directly regulates *FST* gene expression, firstly, we constructed and verified the *STAT3* expression vector, pCMV-Myc-STAT3 by western blotting (Figure 1B). Subsequently, dual-luciferase reporter assays were performed. The promoter reporter gene assay showed that, as expected, both pGL3-basic and pGL3-FST(−340/−980), as a negative control, had very lower luciferase activity, and no difference in luciferase activity was observed between them (*P* > 0.05, Figure 1C). The luciferase activities of pGL3-FST(−980/−340) and pGL3-FST(−980/−340)-mutSTAT3 were 3.39- and 6.23-fold, respectively, higher than that of pGL3-basic (*P* < 0.05, Figure 1C). Moreover, the luciferase activity of pGL3-FST(−980/−340)-mutSTAT3 was significantly higher than that of pGL3-FST(−980/−340) (*P* < 0.05, Figure 1C). These data suggest that the −980/−340 region has promoter activity and that STAT3 inhibits sheep FST promoter activity.

Further co-transfection analysis showed that the luciferase activity of pGL3-FST(−980/−340) was significantly reduced by 22.83% in the cells co-transfected with pCMV-Myc-STAT3, as compared with the cells co-transfected with pCMV-Myc (*P* < 0.05, Figure 1D). Consistent with the above mutation analysis result (Figure 1C), this result also supports that STAT3 inhibits sheep FST promoter activity.

Furthermore, to test whether STAT3 directly regulates sheep *FST* promoter, the pGL3-FST(−980/−340) and either pCMV-Myc-STAT3 or pCMV-Myc were co-transfected into HEK293T cells, and chromatin immunoprecipitation (ChIP) assay was employed with anti-Myc antibody or mouse IgG (negative control). The ChIP-qPCR results exhibited that the *FST* promoter fragment (−547/−356) was significantly enriched (6.16, 20.55, and 8.89-fold, respectively) in the DNA immunoprecipitated by the anti-Myc antibody related to negative controls (mouse IgG, A and B) (*P* < 0.05, Figure 1E). Consistent with the ChIP-qPCR results, agarose gel electrophoresis analysis showed that, compared with negative controls (mouse IgG, A, and B), more PCR products (−547/−356 region of *FST* promoter) were obtained from the DNA fragments immunoprecipitated by the anti-Myc antibody (Figure 1F). In summary, these data indicated that STAT3 directly binds to and negatively regulates the *FST* promoter.

### STAT3 and FST Have Opposite Effects on Cell Proliferation

**FIGURE 2:**
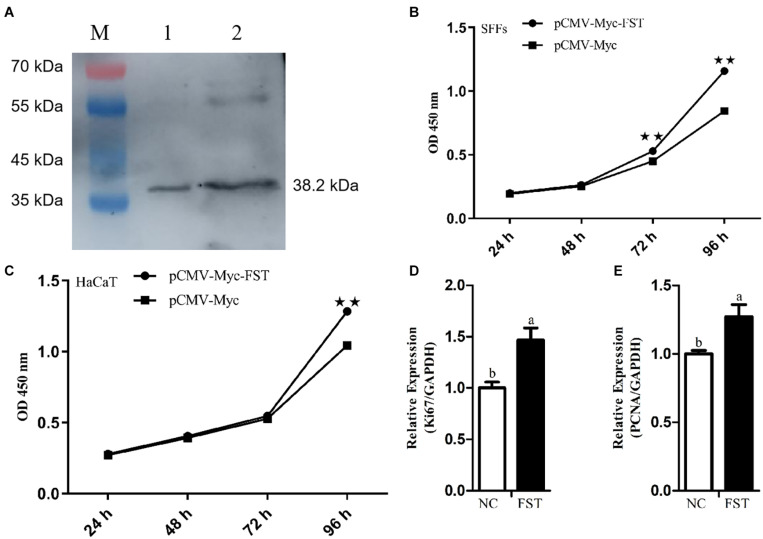
Effects of *FST* overexpression on cell proliferation. **(A)** Western blot recognition of the *FST* expression vector (pCMV-Myc-FST). Lanes 1 and 2: the lysate of the cells transfected with pCMV-Myc-FST (38.2 kDa). **(B,C)** Effects of *FST* overexpression on the proliferation of SFFs and HaCaT cells. **(D,E)** Expression of *Ki67* and *PCNA* in the SFFs transfected with pCMV-Myc-FST. Fold change was calculated referring to the expression of the SFFs transfected with pCMV-Myc at 48 h. All data are representative of three independent experiments and display as the mean ± SEM. For each figure layer, statistical significance was indicated by *P < 0.05, **P < 0.01, and different letters above error bars indicated a statistical significance (*P* < 0.05).

**FIGURE 3:**
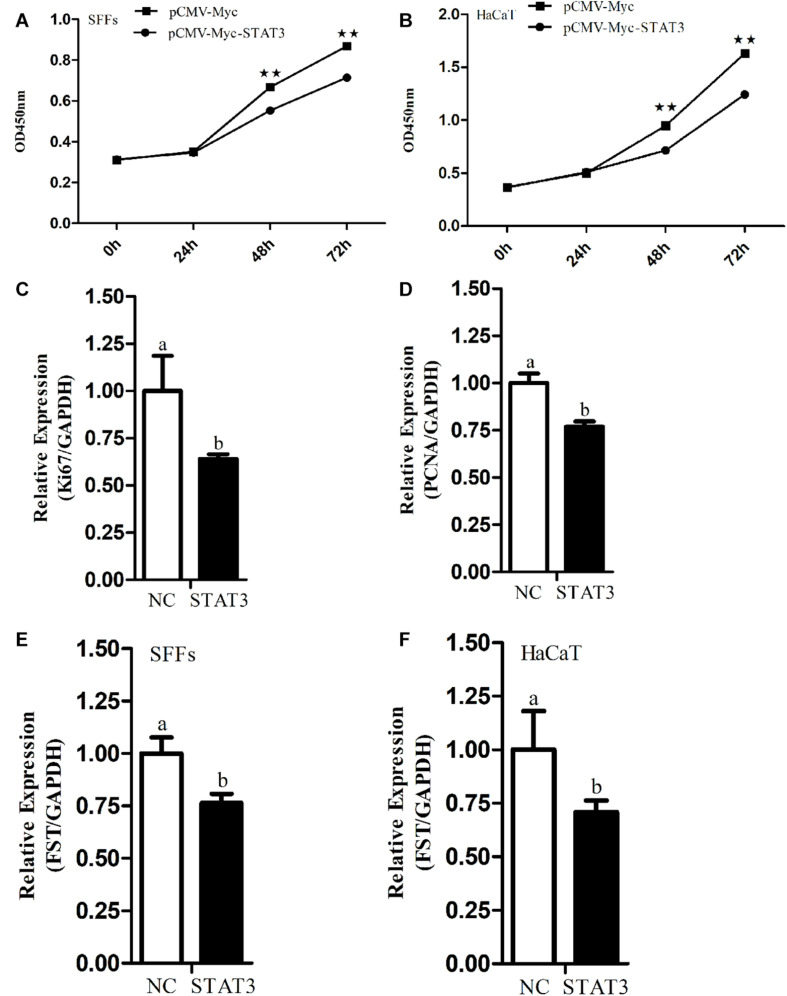
Effects of *STAT3* gene overexpression on cell proliferation and endogenous *FST* expression. **(A,B)** Effect of *STAT3* overexpression on the proliferation of SFFs and HaCaT cells. **(C,D)** Expression levels of *Ki67* and *PCNA* in the SFFs transfected with pCMV-Myc STAT3. **(E,F)** Effects of STAT3 overexpression on the endogenous expression of *FST* gene in SFFs and HaCaT cells. Fold change was calculated referring to the expression of the SFFs transfected with pCMV-Myc at 48 h. All data are representative of three independent experiments and display as the mean ± SEM. For each figure layer, statistical significance was indicated by *P < 0.05, **P < 0.01, and different letters above error bars indicated a statistical significance (*P* < 0.05).

To test whether FST mediates the roles of STAT3 in cell proliferation, we constructed and confirmed the *FST* expression vector (pCMV-Myc-FST) by western blotting (Figure 2A), and investigated the effects of overexpression of *STAT3* and *FST* on cell proliferation using the CCK-8 assay. The results showed that the absorbance of both the SFFs and HaCaT cells transfected with pCMV-Myc-FST was significantly higher than those transfected with pCMV-Myc at 96 h of transfection (*P* < 0.01, Figures 2B,C), suggesting that *FST* promotes the proliferation of SFFs and HaCaT cells. In contrast, the absorbance of both the SFFs and HaCaT cells transfected with pCMV-Myc-STAT3 was significantly lower than those transfected with pCMV-Myc at 48 h and 72 h (*P* < 0.01, Figures 3A,B), suggesting that STAT3 represses the proliferation of SFFs and HaCaT cells. Consistently, *FST* overexpression significantly promoted *Ki67* and *PCNA* expression in the SFFs (*P* < 0.05, Figures 2D,E), while *STAT3* overexpression significantly inhibited *Ki67* and *PCNA* expression in the SFFs, compared with the cells transfected with pCMV-Myc at 48h (*P* < 0.05, Figures 3C,D). Further gene expression analysis showed *STAT3* overexpression significantly reduced the endogenous *FST* expression in both SFFs and HaCaT cells by 76.39 and 71.36%, respectively, compared with the cells transfected with pCMV-Myc at 48 h (*P* < 0.05, Figures 3E,F).

## Discussion

In this study, we revealed that STAT3 directly negatively regulates sheep *FST* gene. Our evidence is as follows: (1) Bioinformatics analysis showed that *FST* promoter harbored a conserved putative STAT3 binding site (Figure 1A). (2) The luciferase reporter assay showed that mutation of STAT3 binding site led to an increase in the *FST* promoter activity and that STAT3 inhibited the *FST* promoter activity (Figures 1C,D). (3) The ChIP-qPCR assay showed that STAT3 directly bound to the *FST* promoter (Figures 1E,F). (4) Further functional analysis showed that *FST* and *STAT3* overexpression had opposite effects on the proliferation of SFFs and HaCaT cells (Figures 2, 3) and that *STAT3* overexpression inhibited the endogenous *FST* expression in SFFs and HaCaT cells (Figures 3E,F). Moreover, Many target genes of STAT3 have been identified, such as forkhead box L2 (*FOXL2*) (Han et al., 2017), interleukin 17A (*IL-17A*) (Kunkl et al., 2019), interferon regulatory factor 4 (IR*F-4*), and B cell lymphoma 6 (*Bcl-6*) (Chen et al., 2019). To our knowledge, for the first time, we demonstrated that *FST* is a bona fide target gene of STAT3 and that STAT3 directly negatively regulates the *FST* gene and inhibits cell proliferation.

In the present study, the bioinformatics analysis showed that besides STAT3, several transcription factors had their binding sites in sheep *FST* gene promoter, such as HOXA4, E2F2, and HNF4. Previous studies have demonstrated that HOXA4 and E2F2 were involved in the development of epidermis and dermis, as well as hair follicles (Stelnicki et al., 1998; Lorz et al., 2010). To better understand the transcriptional regulation of the *FST* gene in sheep hair follicles, it is worth investigating the regulation of the sheep *FST* gene by these predicted transcription factors as well.

In the present study, we found that the STAT3 negatively regulated *FST* gene and inhibited cell proliferation (Figures 1–3). Considering that transcription factors have numerous target genes, we cannot eliminate the probability that STAT3 inhibits cell proliferation partly by regulating the expression of its other target genes. Interestingly, a partial inhibitory repercussion of STAT3 on the promoter activity of pGL3-FST(−980/−340)-mutSTAT3 was observed, as compared with the cells co-transfected with pCMV-Myc (*P* < 0.05, Figure 1D). This may be dual for several reasons. Firstly, STAT3 may bind to its non-canonical binding sites in sheep *FST* promoter and inhibit *FST* promoter activity. Secondly, STAT3 may indirectly regulate *FST* promoter activity through regulation of the expression of the transcription factors which have binding sites in the *FST* promoter. Lastly, STAT3 may indirectly regulate *FST* promoter activity by interaction with some transcription factors, which have binding sites in the *FST* promoter. Further study is required to determine the precise mechanism underlying the partial inhibitory effect of STAT3 on the reporter pGL3-FST(−980/−340)-mutSTAT3 in the future.

In the present study, we demonstrated that sheep *FST* overexpression promoted SFFs and HaCaT cell proliferation (Figures 2B–E). In agreement with our results, it has been shown that FST promotes the proliferation of duck primary myoblasts (Li et al., 2014). Moreover, *FST* overexpression promoted satellite cell proliferation and stimulated muscle fiber hypertrophy in mice (Gilson et al., 2009) and duck (Liu et al., 2012). The knock-down of *FST* significantly reduced the proliferation of the immortalized ovarian surface epithelial and human ovarian carcinoma cell line SKOV3 (Karve et al., 2012). Previous studies showed *STAT3* overexpression inhibited the proliferation of mouse leukocyte and hepatocyte *via* inhibiting *cyclin D* expression (Lee et al., 2002; Matsui et al., 2002), as well as chondrogenic cell line ATDC5 (Suemoto et al., 2007). In agreement, our results showed that *STAT3* overexpression inhibited the proliferation of SFFs and HaCaT cells (Figures 3A–D). However, it has been shown that *STAT3* overexpression has been shown to promote human breast cancer (Bromberg et al., 1999; Ma et al., 2020) and thyroid carcinoma (Kong et al., 2019) cell proliferation. The different effects of STAT3 overexpression on cell proliferation suggest that STAT3 may play different roles in cell proliferation, depending on cell type, cellular context, and species.

Accumulating evidence has demonstrated that STAT3 and FST function in hair follicle morphogenesis and development. STAT3 activation is a prerequisite for the early anagen of hair follicles (Sano et al., 2008) and keratinocytes-specific STAT3 knockout mice exhibited impaired hair cycle (Sano et al., 1999). Additionally, STAT3 can maintain keratinocyte stem/progenitor cell homeostasis *via* facilitating the maturation of the bulge region in mouse hair follicle development (Sano et al., 2000, 2008; Rao et al., 2015; Shibata et al., 2015; Nelson et al., 2016). FST promotes hair follicle development *via* binding activins and preventing the activation of activin receptors (Mcdowall et al., 2008). FST knockout mice displayed thin and curlier vibrissae (Matzuk et al., 1995; Nakamura et al., 2003), and FST transgenic mice exhibited smaller hair follicles and rough and irregular pelage (Wankell et al., 2001). Our previous study showed that sheep *FST* gene polymorphisms were associated with wool quality traits (Ma et al., 2017). Given these previous reports and our previous and present results, we hypothesize that STAT3 controls sheep hair follicle development at least in part *via* direct negative regulation of *FST* expression. Considering the STAT3 binding site in *FST* promoter are conserved across different species, we presume that our results may not be limited to sheep.

There are several limitations in the present study. First, SFFs and HaCaT cell lines were used for transcriptional regulation and function of sheep STAT3 and FST in hair follicle morphogenesis and development. These two cell lines do not originate from hair follicles and the HaCaT cell line is a non-sheep cell line. Both of these two cell lines may not be the best *in vitro* model for our study. However, hair follicles consist of mesenchymal cells and epithelial cells. SFFs are a type of mesenchymal cells (Martin, 1997; Haniffa et al., 2009), and HaCaT cells, a spontaneously immortalized, human keratinocyte line, represent epithelial cells (Deyrieux and Wilson, 2007; Wilson, 2014). These two types of cell lines may reflect some extent the *in vivo* situation of hair follicles. Additionally, these cell lines are widely used to study hair follicle morphogenesis and development (Inui et al., 2000; Ahmed et al., 2011; Luanpitpong et al., 2011; Nakamura et al., 2014; Koobatian et al., 2015). Second, only a 3-kb promoter fragment upstream of the sheep *FST* gene was used for promoter analysis, and the distal promoter region of the sheep *FST* gene was not investigated. Third, only the *in vitro* study was performed in our study. An *In vivo* study needs to be carried out to investigate the regulation of FST by STAT3 in sheep hair follicle development and wool trait formation. Nevertheless, even though there are several limitations in our present study, our results suggests that STAT3 regulates *FST* gene in sheep.

## Conclusion

In summary, in the present study, we closed the genomic gap upstream of sheep genomic *FST* gene (Accession No. MT917184) and demonstrated that STAT3 inhibits the proliferation of SFFs and HaCaT cells at least in part *via* direct negative regulation of *FST* gene expression. Our findings will contribute to an understanding of the *FST* transcriptional regulation and the molecular mechanisms underlying hair follicle development. To gain a better understanding of the mechanisms underlying sheep hair follicle development and morphogenesis, *in vivo* studies will be needed to validate the regulatory relationship between STAT3 and FST in sheep hair follicle development.

## Data Availability Statement

The datasets presented in this study can be found in online repositories. The names of the repository/repositories and accession number(s) can be found in the article/Supplementary Material.

## Ethics Statement

The animal study was reviewed and approved by all animal works were conducted according to the guidance for the care and use of experimental animals established by the Ministry of Science and Technology of the People’s Republic of China (Approval number: 2006-398) and approved by the Laboratory Animal Management Committee of Northeast Agricultural University.

## Author Contributions

NW designed the study and provided funding support. HX and GM carried out the experiments, analyzed data, and wrote the first draft of the manuscript. FM and BN contributed to the subject discussion. HL and NW critically revised the manuscript. All authors reviewed and approved the final version of the manuscript.

## Conflict of Interest

The authors declare that the research was conducted in the absence of any commercial or financial relationships that could be construed as a potential conflict of interest.

## Footnotes

1 http://genome.ucsc.edu/

2 https://mulan.dcode.org/

## Supplementary Material

Supplementary Figure 1 Determination of the genomic gap upstream of sheep *FST* gene by genome walking. The three reverse primers, *FST*-SP1, *FST*-SP2, and *FST*-SP3 were designed according to the published sequences (GCF_002742125.1) on the NCBI website, and three forward primers, ZFP2, ZSP1, and ZSP2 were provided by KX Genome Walking Kit (Zoman Biotechnology, China). The primer pairs ZFP2/*FST*-SP1, ZSP1/*FST*-SP2, and ZSP2/*FST*-P3 were used to conduct the first-, second-, and third-round PCRs for closing the genomic gap upstream of sheep *FST* gene according to the manufacturer’s directions (more detailed information was shown in “MATERIALS AND METHODS”).

Supplementary Table 1 Primers used in this study.

Supplementary Table 2 Original data.
