# Supplementary figures and images for: STAT3 Partly Inhibits Cell Proliferation via Direct Negative Regulation of FST Gene Expression
Source: Front Genet. 2021 Jun 22;12:678667. doi: 10.3389/fgene.2021.678667 (PMC8259742; doi:10.3389/fgene.2021.678667)

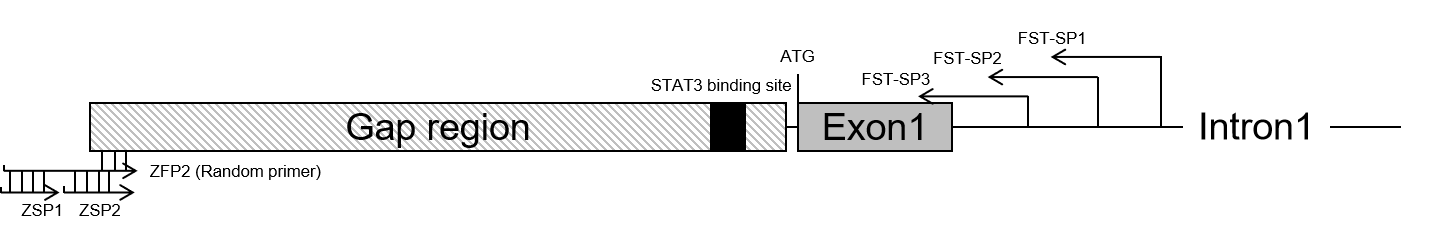

Supplement: Supplementary Figure 1 — Determination of the genomic gap upstream of sheep FST gene by genome walking. The three reverse primers, FST-SP1, FST-SP2, and FST-SP3 were designed according to the published sequences (GCF_002742125.1) on the NCBI website, and three forward primers, ZFP2, ZSP1, and ZSP2 were provided by KX Genome Walking Kit (Zoman Biotechnology, China). The primer pairs ZFP2/FST-SP1, ZSP1/FST-SP2, and ZSP2/FST-P3 were used to conduct the first-, second-, and third-round PCRs for closing the genomic gap upstream of sheep FST gene according to the manufacturer’s directions (more detailed information was shown in “MATERIALS AND METHODS”). [file Image_1.TIF]
