# Supplementary material for: STAT3 Partly Inhibits Cell Proliferation via Direct Negative Regulation of FST Gene Expression
Source: Front Genet. 2021 Jun 22;12:678667. doi: 10.3389/fgene.2021.678667 (PMC8259742; doi:10.3389/fgene.2021.678667)
Supplement: Supplementary Table 1 — Primers used in this study. [file Data_Sheet_1.doc]

| Primer name | Sequence (5’→3’) | Tm/°C | Product/bp | Application |
| --- | --- | --- | --- | --- |
| FST-SP1 | AGCATCCACTAGGCAGAAAGCTA | 60.5 | 3704 | For *FST* promoter |
| FST-SP2 | TTCAGGCTACCGATGCTTATCCC | 64.6 | 3504 |  |
| FST-SP3 | GCTAAGTTGGTCCAACATCTTCCGA | 65.8 | 3288 |  |
| FST-V-F | G*GAATTC*TGGCCCGTCCTAGGCACCAGC | 61.3 | 1051 | For pCMV-Myc-FST |
| FST-V-R | CCG*CTCGAG*ATTACCACTCTAGAATGGAAGATATAGGA |  |  |  |
| *FST*-Q-F | GCCGAATGAACAAGAAGAACAAAC | 54 | 345 | For *FST* real time PCR |
| *FST*-Q-R | TCAGGTGACAGGCACTGGGGTA |  |  |  |
| FST-P(+)-F | atttctctatcgata*GGTACC*CATTTTTGTTTGCTTC | 63 | 640 | For pGL3-FST(-980/-340) |
| FST-P(+)-R | cagtaccggaatgcc*AAGCTT*AATCAGTGACATTTC |  |  |  |
| FST-P(-)-F | cagtaccggaatgcc*AAGCTT*CATTTTTGTTTGCTTC | 63 | 640 | For pGL3-FST(-340/-980) |
| FST-P(-)-R | atttctctatcgata*GGTACC*AATCAGTGACATTTC |  |
| FST-M-F | ATAAGAACTCGGCGA*GGTA*CCTCCTGCCG | 58 | 5430 | For pGL3-FST(-980/-340)-mutSTAT3 |
| FST-M-R | *TACC*TCGCCGAGTTCTTATTTGCGTAGGA |  |  |  |
| FST-C-F | CGTGCAGGACTTGAAGTGGGTG | 60 | 192 | For ChIP qRT-PCR |
| FST-C-F | CACCTTCTGAAACCCTTCCCAAT |  |  |  |
| *GAPDH*-Q-F | CTGACCTGCCGCCTGGAGAAA | 56 | 149 | For *GAPDH* real time PCR |
| *GAPDH*-Q-R | GTAGAAGAGTGAGTGTCGCTGTT |  |
| *Ki67*-Q-F | AGGATGGAAGCAAGTCACCTGGAT | 60 | 129 | For *Ki67* real time PCR |
| *Ki67*-Q-R | CTTCTGAACGGGGACTGGAATCTT |  |  |  |
| *PCNA*-Q-F | CGTCTCATGTCTCCTTGGTGCA | 59 | 104 | For *PCNA* real time PCR |
| *PCNA*-Q-R | GGACATGCTGGTGAGGTTCA |  |  |  |
| STAT3-V-F | TGAAT*GTCGAC*GTATTGTTCTAAAGTGGGT | 52 | 2536 | For pCMV-Myc-STAT3 |
| STAT3-V-R | *GGTACC* TTGGCGCATCCGTCAT |  |  |  |

**Supplementary Table 1 |** Primers used in this study

Note: The underlined and italic type nucleotides are the *EcoR* I and *Xho* I restriction enzyme site for FST-V primer pairs, *Kpn* I and *Hind* III restriction enzyme site for FST-P(+) and FST-P(-) primer pairs, Sal I and Kpn I restriction enzyme site for STAT3-V primer pairs, and directional mutational site for FST-M primer pairs, respectively. The lowercase part is the homologous sequence with pGL3-basic for FST-P(+) and FST-P(-) primer pairs.
